# Amyotrophic lateral sclerosis: the complex path to precision medicine

**DOI:** 10.1007/s00415-018-8983-8

**Published:** 2018-07-27

**Authors:** Kevin Talbot, Emily Feneberg, Jakub Scaber, Alexander G. Thompson, Martin R. Turner

**Affiliations:** Nuffield Department of Clinical Neurosciences, John Radcliffe Hospital, University of Oxford, Oxford, OX3 9DU UK

**Keywords:** Amyotrophic lateral sclerosis, TDP-43, Frontotemporal dementia, Precision medicine

## Abstract

Amyotrophic lateral sclerosis (ALS) is a progressive neurodegenerative disease of the corticomotorneuronal network responsible for voluntary movement. There are well-established clinical, genetic and pathological overlaps between ALS and frontotemporal dementia (FTD), which together constitute the ‘TDP-43 proteinopathies’. An ever-expanding list of genes in which mutation leads to typical ALS have implicated abnormalities in RNA processing, protein homoeostasis and axonal transport. How these apparently distinct pathways converge to cause the characteristic clinical syndrome of ALS remains unclear. Although there are major gaps in our understanding of the essential nature of ALS pathophysiology, the identification of genetic causes in up to 15% of ALS patients, coupled with advances in biotechnology and biomarker research provide a foundation for approaches to treatment based on ‘precision medicine’, and even prevention of the disease in pre-symptomatic mutation carriers in the future. Currently, multidisciplinary care remains the bedrock of management and this is increasingly being put onto an evidence-based footing.

## ALS is a complex multisystem degenerative disease overlapping with frontotemporal dementia

The clinical definition of amyotrophic lateral sclerosis (ALS) has not changed substantially since the first descriptions in the nineteenth century. The essential features of painless, progressive loss of function with evidence of upper and lower motor neuron signs in the same anatomical territory, associated with normal imaging and a supportive EMG, make the diagnosis straightforward in most cases. However, a key indication that ALS is a biologically complex entity is that mutations in more than 20 genes can lead to symptoms and signs which are clinically indistinguishable from sporadic cases. The majority (> 95%) of both familial and sporadic cases of this uniformly fatal disease are characterised at autopsy by TDP-43 proteinopathy (nuclear clearing and cytoplasmic aggregation), but a substantial minority (e.g. due to *SOD1* or *FUS* mutations) do not show immunostaining for TDP-43 [1, 2]. Thus, a picture emerges of clinical, pathological and aetiological heterogeneity, in which ALS is now best conceptualised as a clinical syndrome caused by a number of discrete, but overlapping, biological processes. Although much research continues to focus on the cellular mechanisms defining ALS, as with other neurodegenerative diseases, a general feature of which is compartmentalised pathology, it remains unclear why the motor system is selectively vulnerable. A plausible overarching model, by analogy with cancer, is that ALS arises as a consequence of multiple biological ‘hits’, some of which are genetic, others acquired during development and with ageing [3]. The final common pathway on which these elements converge is unlikely to be a single biochemical or cellular network, but rather the corticomotorneuronal system itself. Understanding how such network vulnerability interacts with genotype, CNS development, environmental exposures and ageing will be key to identifying and applying disease-modifying therapies.

The initiation and control of voluntary motion require a precisely organised top-down connected architecture which has undergone significant expansion in recent primate evolution [4]. The corticospinal tract and its associated connections have some distinctive features, including an expanded number of fibres in humans compared to other primates and a greater number of monosynaptic connections, notably for fine motor control in the upper limb [5]. It has been repeatedly noted that two areas which are relatively spared in ALS, the oculomotor nuclei and the motor neuron pools innervating sphincter function, only connect with higher cortical centres via interneurons. Given the complexity of influences on spinal motor neurons, which as part of the ‘motor unit’ act as the final common pathway of voluntary movement, it is difficult to draw any simple unifying conclusion about whether ALS is a cortically driven disease in all cases. However, even lower motor neuron predominant patients demonstrate pathology in the corticospinal tract at autopsy, suggesting that classifying ALS as a ‘neuromuscular disease’ fails to capture the essential nature of the problem.

A further level of clinical, genetic and pathological heterogeneity is manifest in the overlap between ALS and frontotemporal dementia (FTD). 40–50% of patients presenting with isolated FTD have TDP-43 pathology at autopsy [6]. In turn, ALS patients display a spectrum of cognitive impairment ranging from frank behavioural variant FTD in 3–5% to more minor or sub-clinical forms of loss of executive function in up to 50% [7]. Thus, extension beyond the corticomotorneuronal network is not inevitable in ALS, which for about half of patients remains restricted to motor function. Whether the pathological overlap in the TDP-43 proteinopathies reflects propagation through interconnected cerebral networks or a multifocal process is unknown.

Studies of environmental exposures leading to ALS have so far been unconvincing. Geographical variation, over and above that explained by genetic founder effects, is minimal, with all populations studied to date using rigorous methods having a similar incidence (around 2/100,000 population per year) and prevalence (5–7/100,000). However, studies using unbiased population registers have only been carried out in populations of European genetic heritage, and there are major gaps in epidemiological research in ALS. One intriguing observation is an apparently lower rate of ALS in Africans. In Cuba, a lower frequency of ALS is seen in sub-populations with a greater African genetic heritage compared with those of European descent [8]. If correct, this might suggest that some of the complex genetic susceptibility to ALS was acquired after the migration of the ancestral human population which gave rise to all modern non-Africans, around 70–80,000 years ago. Although questionnaire-based epidemiology studies are subject to significant recall bias, evidence of an association between trauma and ALS is accumulating [9]. For as yet unexplained reasons, people with ALS may have a distinct metabolic profile, with subgroups displaying a hypermetabolic state, greater weight loss and a worse prognosis [10]. Further observations to support the hypothesis that ALS patients may be metabolically distinct are that higher cholesterol levels at diagnosis are associated with longer survival [11, 12]. Relatives of people dying of ALS may have a lower overall rate of heart disease, perhaps suggesting that the complex genetic profile underlying ALS risk confers cardiovascular protection [13].

## The genetic contribution to ALS provides a window on pathogenesis

The majority of ALS patients do not have a family history of the condition and it is therefore considered to be principally a ‘sporadic’ disease. Familial ALS accounts for up to 10% of all incident cases, and the genetic mutation responsible has now been explained in 70–80% of familial cases, depending on the population studied, with mutations in over 20 different genes, mostly in an autosomal dominant inheritance pattern [14]. Importantly, mutations in these genes are also found in a substantial minority of patients without a family history, indicating that they can also act as incompletely penetrant rare variants of significant disease determining effect. Overall, first-degree relatives of patients with ALS have a lifetime risk of 1.1% in large population studies, and a relative risk of 2.2–6.9 compared to the general population, in which the lifetime risk of ALS is approximately 0.3% [15, 16]. In comparison to other complex diseases like schizophrenia, the contribution to ALS causation of common variants as revealed by genome-wide association studies appears modest, though larger studies are awaited [17]. Thus, ALS is a disorder in which a genetic contribution is likely to be present in most patients, but with a complex spectrum of effect sizes ranging from classical Mendelian patterns of inheritance to multiple rare variants acting in combination, in both cases with environmental and stochastic effects providing the age-dependant subsequent steps required for disease manifestation.

The proteins encoded by ALS-related genes can be grouped into a few distinct pathways based on their known function. Although this has been instrumental in increasing understanding of the molecular basis of ALS, proteins in the nervous system frequently display specialised or adaptive functions, and mutation-induced toxicity may be through an unrelated mechanism. For example, mutations in *SOD1*, the first genetic cause of ALS to be identified, do not appear to cause neurodegeneration by interfering with the canonical enzymatic function of the protein but by producing an acquired, as yet unexplained, toxicity via abnormal protein homeostasis [18]. In contrast, there are examples of ALS-causing mutations, occurring in a minority of cases and typically with a lower motor neuron phenotype, such as in dynactin subunit 1 [19], the actin-binding protein profilin-1 [20], the microtubule subunit TUBA4A [21] and the kinesin motor protein KIF-5A [22], which point to cytoskeletal and axonal defects as a contributor to ALS pathology and which have a more obvious relationship to the characteristic function of the protein.

A major advance in our understanding of cellular mechanisms in ALS came from the identification of causative mutations in the *TARDBP* gene [23], coding for TDP-43, a protein found in the pathological aggregates in motor neurons in the majority of cases of ALS [24]. Although related to TDP-43 through membership of the general class of proteins involved in RNA processing, mutations in the *FUS* gene lead to ALS with FUS, not TDP-43, pathology [1]. Both proteins belong to the heterogeneous ribonucleoprotein protein family (hnRNP), possessing RNA recognition motifs, and are involved in multiple steps of RNA processing, such as splicing, RNA transport and miRNA biogenesis [25]. Subsequently, rarer mutations have been identified in ALS in a range of other ribonuclear proteins including hnRNPA1 [26] and Matrin 3 [27]. Given the complexity and diversity of RNP function in neurons, the question arises whether there is a single aspect of RNA handling in neurons which is most relevant to ALS. RNPs typically contain so-called ‘low complexity domains’ which allow hnRNP–RNA complexes to undergo liquid–liquid phase transition in the formation of membraneless organelles (including stress granules and P-bodies) in which protein translation is stalled, conserving cellular function during stress [28]. This property of RNPs, however, may also promote fibrillarisation of the proteins if the dynamic balance is disturbed by age-related failure of protein homeostasis or by a mutation [29]. The recent identification of mutations in the stress granule protein TIA-1 adds further weight to the role of abnormal stress granules assembly and dynamics in ALS [30].

Mutations in the autophagy adaptors optineurin (*OPTN*), ubiquilin 2 and p62 (*SQSTM1*) found in ALS point to defects in the aggrephagy pathway, a cargo-specific form of autophagy, and suggest defects in protein homeostasis may contribute significantly to disease pathogenesis [31]. Impaired autophagy is also implicated by the discovery of ALS-causing mutations in *TBK1*, a protein kinase required for efficient cargo recruitment [32]. Many of the mutations described are predicted to lead to loss of TBK-1 function, directly implicating impaired autophagy, though missense mutations may exert their effect in a different way. The fact that stress granule assembly can be regulated by protein chaperones via the autophagosome provides a potential link between mutations in RNA-interacting proteins and those in protein quality-controlling genes [33].

The pathogenic mechanism of the most common genetic cause of ALS, a hexanucleotide repeat expansion (HRE) in an intron of the gene *C9orf72*, is debated, with contributions potentially from several mechanisms [34, 35]. Loss of function by epigenetic silencing of *C9orf72* expression by the HRE has been postulated to impair vesicular transport [36] and also autophagy [37]. However, there is also compelling evidence that mutation-induced toxicity, acting through RNA repeat aggregates, sequestrates hnRNPs and leads to a number of deleterious effects including nucleolar stress [38]. Non-ATG translation of the intronic repeat results in the production of cytoplasmic dipeptide repeats (DPRs), which have also been shown to exert toxicity through disruption of the normal assembly and regulation of membraneless organelles [39].

Despite the lack of comprehensive pathophysiological explanations by which any of the ALS causing mutations cause motor system degeneration, a number of common themes are emerging (stress granule dynamics, protein quality control) which may serve as plausible therapeutic targets. How mutations in these seemingly separate pathways all cause specific degeneration of the motor tract with or without frontotemporal dementia is unclear. Changes in cellular metabolism due to the cumulative effects of ageing undoubtedly contribute to pathology, as the majority of these mutations are tolerated for many decades without being detrimental to function, in contrast to the often severe and acute toxicity seen in model systems. It remains unclear, however, if the onset of ALS is caused by a multifocal decompensation of the motor system or if a stochastic event in a single motor neuron pool acts as a ‘seed’ from which a pathological cascade then propagates.

## Advances in biomarkers

Objective markers of disease activity are needed to facilitate early diagnosis, to predict disease progression and provide a more rapid therapeutic readout to streamline clinical trials. ALS is a clinical diagnosis and, with the exception of neurophysiology, ancillary investigations like neuroimaging and neurochemical biomarkers are not included in the diagnostic criteria which have been developed to date. Electromyography is used to define the degree of clinical certainty in the revised El Escorial criteria for ALS and probably modestly increases diagnostic sensitivity [40]. Conventional clinical-grade MRI of the brain is typically normal in ALS; however, more sophisticated structural MRI studies show disease-related changes within the corticospinal tracts, interhemispheric white matter projections and primary motor cortices [41, 42]. Longitudinal MRI studies suggest that a decrease in motor and extramotor grey matter volume may be sensitive to disease progression in ALS [43], and that cortical changes may occur even years before disease onset in *C9orf72* genetic mutation carriers [44]. However, more subtle pre-symptomatic changes in brain function may ultimately prove more sensitive outcome measures for future neuroprotective strategies [45, 46].

Neurochemical biomarkers, either disease-specific proteins or more generic markers of the neurodegenerative process, might help provide evidence of target engagement in clinical trials as well as evidence of therapeutic effects in reducing disease activity. Neurofilament light chain and phosphorylated heavy chain, non-specific markers of axonal damage, are greatly elevated in cerebrospinal fluid (CSF) and blood in ALS and FTD compared to neurological disease controls and disorders which might mimic ALS [47, 48]. Neurofilament levels demonstrate stability over the course of disease, suggesting that they are a promising marker for monitoring response to treatment, given that the absolute level appears robustly correlated to the rate of disease progression [49, 50]. Neurotrophin receptor p75, a motor neuron-specific membrane protein, is upregulated in the spinal cords of mutant SOD1 mice. Its extracellular domain (p75^ECD^) undergoes renal excretion and is increased in the urine of ALS patients compared to controls. Because longitudinal p75^ECD^ levels correlate with progressive reduction in the revised ALS Functional Rating Scale score, it has been proposed as a marker of disease progression [51]. Most recently, a group of microglial proteins of the chitinase family has shown a good correlation with disease progression measured in CSF [52].

TDP-43 levels in ALS, measured in either blood or CSF, are highly variable in studies published to date [24, 53–55]. Future investigations of TDP-43 as a biomarker may focus on detection of disease-specific isoforms (e.g. degradation fragments, oligomers), since commonly available antibodies only detect the physiological form of TDP-43 [56]. Dipeptide repeat proteins present in postmortem material of C9orf72-related ALS and FTD cases have also been detected in the CSF of affected individuals and pre-symptomatic mutation carriers [57]. Dipeptide levels are stable in longitudinal testing, but in vitro experiments with antisense constructs show that extracellular levels are a good reflection of the degree of silencing of the C9orf72 repeat RNA and therefore mark an important step towards the development of pre-clinical and treatment response assays for this important sub-group of ALS-FTD [58].

## Emerging therapies beyond riluzole

Despite several decades and over 100 clinical trials, riluzole has been the sole drug in routine clinical use in ALS and shows only a modest effect in improving survival. Edaravone, a free radical scavenger given by intravenous infusion and used in Japan for the treatment of ischaemic stroke, has recently been approved for ALS in both Japan and the USA. The first randomised double-blind placebo-controlled trial of edaravone in 127 ALS patients did not show a significant difference in the primary end point of change in the revised ALS functional rating scale [59]. Post hoc analysis of this data demonstrated differences in a subgroup of patients with relatively early disease; hence, a further randomized trial was carried out in 137 patients, restricting recruitment to a more well-defined group with symptom onset within 2 years of enrolment, no respiratory symptoms and without severe disability according to the ALSFRS-R score [60]. This reported a statistically significant, though modest, reduction in the decline of ALSFRS-R score (mean change − 5.01 in edaravone vs − 7.5 in placebo) at the end of the 24-week treatment period in the group receiving edaravone. Unfortunately, the trial was not powered to detect an effect in arguably the most important outcome, survival, and due to revisions in the ALSFRS, cannot be compared directly with the effectiveness of riluzole. Although edaravone was well tolerated, the protocol for administration is cumbersome, involving intravenous infusion for 10 days every 4 weeks. Edaravone may therefore present challenges for patients with significant disability and comes with significant treatment costs.

A landmark in molecular therapeutics is the use of antisense oligonucleotides (ASOs) to target specific genes to neutralise the toxic effect of mutations or to alter splicing. The outlook for children affected with spinal muscular atrophy, a childhood motor neuron disorder which in its most severe form is uniformly lethal in infancy, has been transformed by the advent of nusinersen, an ASO which alters the splicing of the SMN2 gene to produce more full length SMN protein [61]. In clinical trials of Type 1 (severe) SMA, children treated at or near diagnosis survived beyond their expected lifespan and acquired unprecedented motor function. Early clinical trials of ASOs in ALS patients carrying specific genetic mutations are now being conducted in those carrying mutations in *SOD1* [62] with ASO trials aimed at neutralising the *C9orf72* hexanucleotide repeat soon to follow. This is a rapidly advancing field in which technological developments such as genome editing [63] may ultimately be employed to treat asymptomatic gene carriers to prevent the onset of disease.

## Advances in clinical care

### Gastrostomy

Gastrostomy insertion to circumvent dysphagia due to ALS has been widely practised for decades with the primary aim of supporting well-being and hydration, though it probably also has a small positive effect on survival. A small pilot study has even suggested that supplementation beyond predicted energy demands is beneficial [64, 65]. More formal trials of active nutrition strategies with food supplementation are ongoing. The optimal timing and method of gastrostomy insertion is debated, especially in patients with significant respiratory involvement. Radiologically inserted gastrostomy (RIG) has been advocated in patients with respiratory compromise, despite a higher rate of local complications and reinsertions using this method. However, with appropriate precautions, PEG may be equally safe in carefully selected high-risk patients through a modified approach [66]. A recent large prospective study of gastrostomy insertion in ALS, comparing RIG, PEG and per-oral radiologically inserted gastrostomy (PIG), demonstrated no difference in mortality between PEG and RIG, including patients with FVC < 50% [67].

### Ventilatory assistance

The use of non-invasive ventilation (NIV) for symptomatic treatment in patients with respiratory dysfunction due to ALS has beneficial effects on survival and quality of life [68]. Studies of the early introduction of NIV (prior to significant deterioration in FVC) have reported conflicting results in terms of survival, although early NIV does appear to slow decline in FVC [69]. Other methods to improve survival and respiratory morbidity of ALS have been studied. Diaphragm pacing, direct stimulation using electrodes inserted into the underside of the diaphragm, has been examined as a means to overcome or delay respiratory failure in a randomized controlled trial [70]. The trial had to be stopped early due to an excess of early deaths in the group assigned to receive diaphragm pacing, raising concerns about the safety and efficacy of this procedure.

A small study of cough augmentation using mechanical insufflation–exsufflation (MI–E), used to aid clearance of respiratory tract secretions to prevent respiratory infections, did not demonstrate a significant difference when compared to a simple breath-stacking technique in terms of frequency or duration of respiratory infections, or mortality [71]. It should be noted though that breath stacking may not be appropriate for all patients, particularly those with severe bulbar impairment.

## Future prospects

Amyotrophic lateral sclerosis is a clinical syndrome with multiple causes. Although it is reasonable to hope that degeneration of motor neurons could be prevented or ameliorated by targeting specific nodal points in cellular pathways which are common to all cases, independent of aetiology, research has so far failed to identify common therapeutic targets. The very significant increase in knowledge of ALS pathogenesis in the last few decades has been driven by genetic discoveries and argues in favour of a model in which diverse biological insults can lead to ALS and in which the point of convergence is the corticomotorneuronal system in all its network complexity, rather than a specific cell type.

Rapid advances in gene-based therapies now offer a clear roadmap to treatment or even prevention for the substantial minority of patients with inherited ALS. The complexity of pathways involved in pathogenesis for the majority of ALS patients without a defined genetic cause, the late presentation of the disease and its widely distributed anatomical basis present a significant challenge, which is likely to require tailored polytherapy. Drug pipelines are currently focussed on pathways (neuroinflammation, autophagy, mitochondrial function) emerging from imperfect pre-clinical models, and which may be downstream of the early mechanistic events in motor neuron degeneration which are intuitively more therapeutically tractable, but which are still to be identified.
